# Coral Luminescence Identifies the Pacific Decadal Oscillation as a Primary Driver of River Runoff Variability Impacting the Southern Great Barrier Reef

**DOI:** 10.1371/journal.pone.0084305

**Published:** 2014-01-08

**Authors:** Alberto Rodriguez-Ramirez, Craig A. Grove, Jens Zinke, John M. Pandolfi, Jian-xin Zhao

**Affiliations:** 1 Radiogenic Isotope Facility, School of Earth Sciences, The University of Queensland, Brisbane, Queensland, Australia; 2 NIOZ Royal Netherlands Institute for Sea Research, Department of Marine Geology, Den Burg, Texel, The Netherlands; 3 School of Earth and Environment, The University of Western Australia and the UWA Oceans Institute, Australia and the Australian Institute of Marine Science, Perth, Western Australia, Australia; 4 Australian Research Council Centre of Excellence for Coral Reef Studies, Centre for Marine Science, School of Biological Sciences, The University of Queensland, Brisbane, Queensland, Australia; University of Connecticut, United States of America

## Abstract

The Pacific Decadal Oscillation (PDO) is a large-scale climatic phenomenon modulating ocean-atmosphere variability on decadal time scales. While precipitation and river flow variability in the Great Barrier Reef (GBR) catchments are sensitive to PDO phases, the extent to which the PDO influences coral reefs is poorly understood. Here, six *Porites* coral cores were used to produce a composite record of coral luminescence variability (runoff proxy) and identify drivers of terrestrial influence on the Keppel reefs, southern GBR. We found that coral skeletal luminescence effectively captured seasonal, inter-annual and decadal variability of river discharge and rainfall from the Fitzroy River catchment. Most importantly, although the influence of El Niño-Southern Oscillation (ENSO) events was evident in the luminescence records, the variability in the coral luminescence composite record was significantly explained by the PDO. Negative luminescence anomalies (reduced runoff) were associated with El Niño years during positive PDO phases while positive luminescence anomalies (increased runoff) coincided with strong/moderate La Niña years during negative PDO phases. This study provides clear evidence that not only ENSO but also the PDO have significantly affected runoff regimes at the Keppel reefs for at least a century, and suggests that upcoming hydrological disturbances and ecological responses in the southern GBR region will be mediated by the future evolution of these sources of climate variability.

## Introduction

Understanding past climate variability and the historical occurrence of extreme weather events, such as tropical cyclones and floods, is critical when predicting the ecological consequences of future climate change as well as preparing for their impacts on human coastal settlements. Although the effects of the El Niño-Southern Oscillation (ENSO) and the Pacific Decadal Oscillation (PDO) on Australian hydrological regimes are relatively well understood [1]–[6], the 2010–2011 La Niña event, one of the strongest on record [7], severely impacted human communities and coastal ecosystems along the Queensland coast of Australia. At the start of 2011, heavy rainfall caused one of the most significant floods in Australia’s recorded history, followed by severe Tropical Cyclone Yasi, which was the strongest cyclone to make landfall in Queensland since 1918 [7]. More recently, by the end of January 2013, ex-Tropical Cyclone Oswald strongly affected human populations along the east coast of Australia due to the extreme rainfall that broke historical records (precipitation and flood) at several localities [8]. Comprehensive historical analyses of such extreme events are scarce because of the lack of long-term instrumental and proxy climate records [9]–[12]. Therefore, predictions of their frequency and intensity remain uncertain.

Natural archives, such as annually-banded coral skeletons, can be used to derive proxy climate data on seasonal to centennial time-scales, extending far beyond instrumental records [13], [14]. For instance, luminescent lines in coral skeletons, which are caused by the incorporation of terrestrial humic acids carried to the reef during flood events [15], are a reliable proxy for reconstructing freshwater inputs to coastal ecosystems and regional precipitation variations [16], [17]. While the use of coral luminescence has increased our understanding of how climatic cycles influence rainfall, flood regimes and hurricane activity [18]–[23], recent advances in luminescence controls and application techniques [24] have revealed previously unidentified relationships with climate phenomena, such as the PDO, contributing to local and regional analyses of past, present, and future climate variability [25].

For the east coast of Australia, ENSO is the dominant driver of inter-annual rainfall variability [26], yet this ENSO–rainfall teleconnection is, in turn, modulated by the Interdecadal Pacific Oscillation (IPO, similar to PDO) [27], [28]. Consequently, historical analysis analyses of rainfall, river discharge and flood risk modelling have identified that during negative/cool PDO phases, the impact of La Niña events on rainfall/floods is greater than during positive/warm PDO phases [6], [29]–[32]. For the GBR, it is known that ENSO events unevenly affect the system but the influence of other large-scale sources of climate variability has not been fully assessed [33]. While some studies on the GBR have verified the relationship between ENSO and coral luminescence [19] and river flow and rainfall reconstructions based on luminescence [17], [34], the modulating effect of the PDO on such records has received little attention. Thus far, varying correlations between river flow reconstructions and ENSO indices using warm (1925–1946) and cold (1947–1976) phases of the PDO confirm the non-stationary ENSO-river flow teleconnection for the GBR [20]. Therefore the nature and extent of the relationship between the PDO and coral luminescence records or luminescence-based rainfall/runoff reconstructions remain poorly constrained for the GBR. Determining primary drivers of inter-annual and decadal luminescence will not only allow better rainfall/runoff reconstructions but also improve the predictability and management of hydrological-related disturbances impacting human populations and reef ecosystems along the GBR catchment area.

Here, we present the first decadal-scale (90-year) composite record (1921–2011) of luminescence spectral ratios from multiple coral colonies as an indicator of the Fitzroy River discharge to the Keppel reefs, southern GBR. We also examine potential environmental (runoff and rainfall) and climatic (ENSO and PDO) drivers of luminescence variability on monthly to multi-decadal time scales and discuss key implications for the hydroclimatology of the southern GBR. Our results support growing indications that the future evolution of ENSO and the PDO will determine the frequency and intensity of extreme climatic events affecting Australia’s east coast (i.e. floods) and provide new insights into the significant role that the PDO cycles play in coral reef dynamics of the southern GBR.

## Methods

### Ethics Statement

Sample collection was conducted under the Great Barrier Reef Marine Park Authority (GBRMPA) permit number G10/33402.1.

### Study Site and Sampling

**Figure 1:**
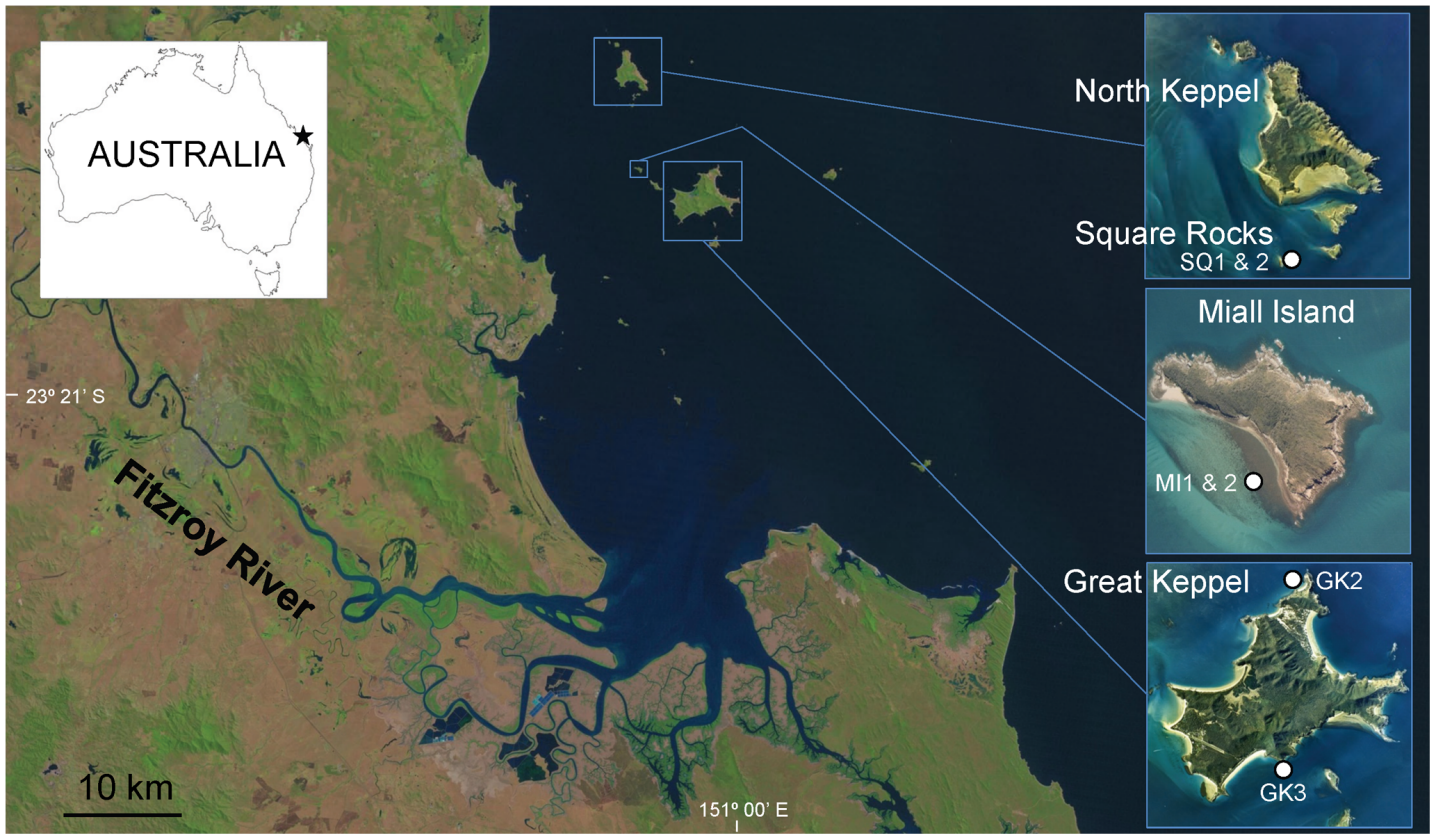
Map displaying the location of the Keppel Islands, sampling sites (white dots) and the Fitzroy River. Additional information about sampling sites is provided in Table S1. Satellite image obtained from http://glovis.usgs.gov/and inset aerial photos courtesy of P. Willams.

Coral cores were collected with a pneumatic drill along the growth axis from six massive *Porites* sp. colonies at water depths between 3–7 m from four locations in the Keppel islands, inshore Great Barrier Reef (23°05′-04′ S and 150°54′-53′ E; Figure 1, Table S1). These continental islands are surrounded by fringing reefs [35] with relatively high coral cover (52%), and are dominated by extensive stands of branching *Acropora* spp [36]. The reefs are influenced by terrestrial run-off from the Fitzroy catchment, the largest seaward-draining catchment discharging to the GBR lagoon with an area of ∼144 000 km^2^ [37], [38]. All sampling sites were located within 50 km of the mouth of the Fitzroy River (Figure 1, Table S1), which is a major source of terrestrial material to the GBR lagoon [39]–[41] delivering more than 3400 ktonnes/yr of total suspended solids, only second to the Burdekin River [42]. The mean annual river discharge measured at the closest gauging station to the river mouth (Rockhampton) is 4.8×10^6^ ML, reaching up to 22×10^6^ ML during large flood events [43], [44]. The climate of the region is characterized by a winter dry season (April to September) and a summer wet season (October to March) [20], [45]. As a result, river discharge mainly occurs in the summer-wet season [41], [46]. Although the Fitzroy River discharges to the south of the Keppel islands, predominant south-east winds and currents promote a north-flowing movement of flood plumes generated during high flow events [47]. Further, the Coriolis Force contributesto diverting flood plumes northwards on the GBR [48].

### Luminescence Analysis

Coral cores were cut longitudinally using a circular saw into 7 mm thick slabs. Before luminescence analysis, coral slabs were X-rayed and then treated with NaOCl for 24 h to remove organic contaminants that can quench the luminescence signal [49]. The slabs were ultrasonically rinsed several times with ultrapure water (18.2.MΩ.cm) and subsequently oven-dried for 24 h (50°C).

The Spectral Luminescence Scanning (SLS) technique was applied to the six coral cores in order to quantify skeletal luminescence [24] and to reconstruct the historical river influence in the Keppel reefs. This method scans coral slabs under a UV light source using a line-scan camera (Figure S1), which records luminescence emission intensities into three spectral ranges, blue (B), green (G) and red (R). RGB intensities were acquired using the Line Scan Software Version 1.6 (Avaatech). The spectral G/B ratio was used as a proxy for river runoff as this relationship normalises the humic acid (G) signal derived from hinterland soils to the skeletal aragonite (B) signal [24], [50].

### Age Model and Coral Composite G/B Record

Age models were constructed by counting density bands using digital X-rays. In addition to X-rays, crossdating, using conspicuous luminescent lines, validated the age models for all cores [19], [24]. Inter-annual chronologies were based on the seasonal cycle of G/B ratios. We assigned the G/B minima values to the driest month (August), according to historical rainfall and river discharge records (see below - Environmental and climatic data). As SLS provides data at sub-weekly resolution [24], G/B time series from each core were linearly interpolated to 12 points per year to obtain monthly chronologies using AnalySeries 2.0 [51].

A composite G/B record spanning 90 years (1921–2011) was created by standardizing the six coral cores by the mean (monthly values) and SD of the time period common to all the cores (1982–2010) and then averaging the standardized records. This procedure reduces the intrinsic variability of individual records and enables the identification of a common regional climatic signal [9], [19], [25], [52]. The agreement between cores was verified by correlating (Pearson linear correlation) G/B monthly and annual anomalies over five common periods for cores with overlapping records (Table S2). Annual anomalies were calculated by averaging all monthly G/B anomalies from August through to July to account for river flow and rainfall extremes during the summer (October-March) in north-eastern Australia [20]. Additionally, the quality of crossdating was assessed by applying a similar approach used for tree-ring chronologies by the program COFECHA [53]. Each individual standardized G/B record was correlated with the average of all other standardized G/B records (the composite record minus the record being tested). A positive and significant correlation indicates that the tested record is crossdated precisely [53].

### Luminescence Drivers

To verify the influence of the Fitzroy River on luminescence variability, the highest flood peaks were plotted against the long-term G/B composite record. To validate the composite G/B record, monthly and annual averages of stream water level, stream discharge, and rainfall (see below - Environmental and climatic data) were correlated with monthly and annual G/B values over the period of 1921 to 2011. In addition, agreement between each individual coral record and environmental dataset was assessed by correlating annual averages of stream water level, stream discharge, and rainfall data with annual G/B anomalies.

To explore potential climatic drivers of luminescence variability, the Southern Oscillation Index (SOI), Niño 3.4, PDO, and CPIPO (combined paleo Interdecadal Pacific Oscillation and Pacific Decadal Oscillation) indices were compared with the long-term G/B composite record using monthly and annual averages over the period 1921 to 2011 (see below Environmental and climatic data). To further examine the temporal variability in luminescence, spectral analysis (REDFIT) [54] was applied to annual G/B anomalies, and correlations between G/B and stream discharge, rainfall, SOI and the PDO index during the Australian climatic seasons were calculated. Monthly values of the G/B anomalies and environmental and climatic variables were averaged accordingly to summer (December to February), autumn (March to May), winter (June to August) and spring (September–November).

Finally, to identify potential drivers explaining most of the variability in the composite record, a distance-based linear model (DISTLM), using a resemblance matrix of G/B (based on Euclidean distance) and the forward procedure, was applied [55], [56]. Forward selection adds one variable at a time to the model, choosing the variable at each step which produces the greatest improvement in the value of the selection criterion. We used adjusted R^2^ as selection criterion instead of R^2^ as we aimed to include only predictor variables that significantly explained the variation in the model. Predictor variables comprised river discharge, rainfall, the SOI and PDO indices. DISTLM outcomes provide a marginal test, fitting each variable individually (ignoring all other variables), and a sequential test, fitting each variable one at a time, conditional on the variables that were already included in the model [55], [57]. Analyses were done using PERMANOVA+ for PRIMER v6.

### Environmental and Climatic Data

Historical flood peaks (m) were obtained from Rockhampton, the nearest gauging station to the Fitzroy river mouth. This manual recording station registers only when the Fitzroy River exceeds a minimum height threshold (http://www.bom.gov.au/hydro/flood/qld/brochures/fitzroy). River flow data (Megaliters/day) and stream water level (m) were obtained from the Queensland Department of Environment and Resource Management gauging station on the Fitzroy River at The Gap (Station number 130005A) and Riverslea (130003A) (http://watermonitoring.derm.qld.gov.au/host.htm), and rainfall (mm) from the Australian Bureau of Meteorology at Pacific Heights (Station number 033077) (http://www.bom.gov.au/climate/data). These river and rainfall data sets provided the longest records for comparative purposes. The SOI and PDO data were obtained from the Australian Bureau of Meteorology (http://www.bom.gov.au/climate/data), and from the Joint Institute for the Study of the Atmosphere and Ocean (http://jisao.washington.edu/pdo/PDO.latest), respectively. The combined paleo Interdecadal Pacific Oscillation (IPO) and PDO index (CPIPO) was obtained from Henley et al. [58]. The Niño 3.4 data was obtained from the Climate and Global Dynamics Division (CGD) of the National Center for Atmospheric Research (http://www.cgd.ucar.edu/cas/catalog/climind/TNI_N34/index.html#Sec5). We will now refer to the PDO and IPO collectively as the PDO-IPO, since they are considered the same broad-scale climatic phenomenon [28], [58]–[60], unless the distinction is necessary.

## Results

**Figure 2:**
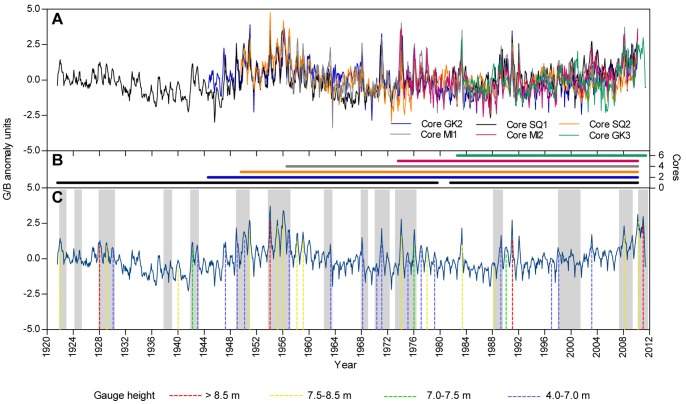
Time series of G/B anomalies for the period 1921–2011. (A) Monthly G/B anomalies for all six cores. (B) Number of cores used to construct the composite record. (C) Long term G/B composite record, with major flood events registered by instrumental records. Colour dashed lines under the profile denote the highest individual flood events registered at Rockhampton, the nearest gauging station to the river mouth (data from Water Division Brisbane, Bureau of Meteorology, Station 039264). Colours refer to gauge height in m. Shaded areas correspond to La Niña periods.

The six cores analysed here showed excellent reproducibility in terms of luminescence (G/B ratios). The six G/B time series showed similar variations over the time period common to all the cores 1982–2010 (Figure 2A). A strong agreement in G/B profiles was also evident, even for the longest coral records (core GK2 and SQ1; Figure 2A). Significant correlations of both monthly and annual G/B anomalies for cores with overlapping records showed the high consistency between cores (Table S3; Table S5–S9). Only one non-significant correlation of annual G/B anomalies was observed between cores SQ2 and MI2 (1973–2010) (Table S6). The correlation was significant, however, when considering monthly data (Table S6). Strong significant correlations were observed between (1) the G/B composite record and each G/B record for the time period common to all the cores (Table 1) and (2) the rest of the time periods with overlapping records (Table S6–S7), confirming that the cores were crossdated correctly and therefore, the common environmental signal (runoff) was optimized.

**Table 1:** Correlation coefficients (R) of monthly and annual G/B anomalies between the composite record and each core from 1982 to 2011.

	Composite record (monthly)		Composite record (annual)	
GK2	**0.75**	p<0.001	**0.83**	p<0.001
SQ1	**0.73**	p<0.001	**0.87**	p<0.001
SQ2	**0.60**	p<0.001	**0.56**	p = 0.002
MI1	**0.81**	p<0.001	**0.91**	p<0.001
MI2	**0.78**	p<0.001	**0.82**	p<0.001
GK3	**0.58**	p<0.001	**0.57**	p = 0.002

Bold values significant at p<0.05.

**Figure 3:**
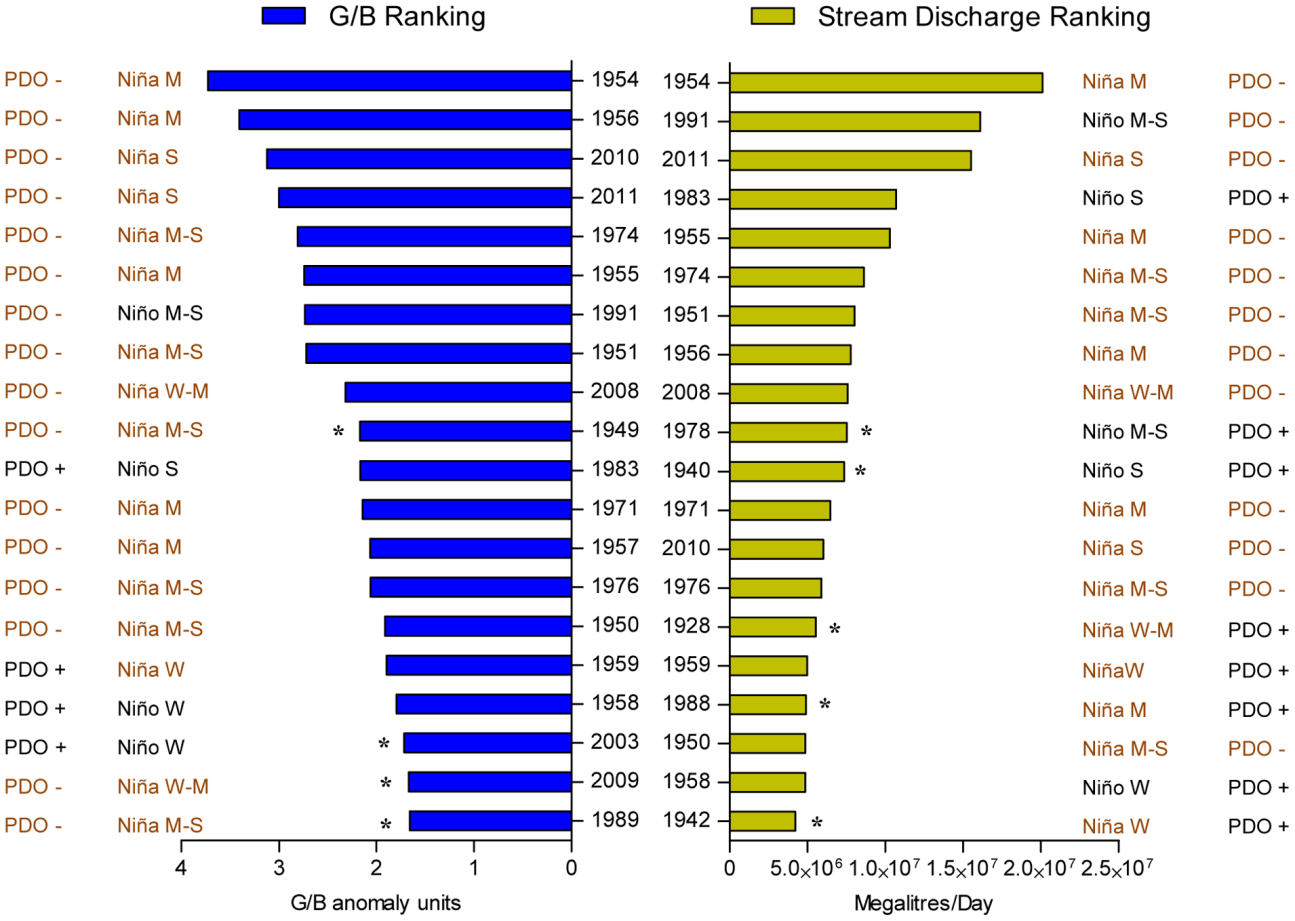
Comparison of the 20 highest G/B anomalies and monthly stream discharge rates for the period 1921–2011. Year, PDO phase (positive/negative) and ENSO state (Niño/Niña) is indicated for each record. Asterisks denote if a record is present only in one ranking. Stream discharge data from Queensland Department of Environment and Resource Management gauging stations on the Fitzroy River at The Gap (Station number 130005A) and Riverslea (130003A) (http://watermonitoring.derm.qld.gov.au/host.htm). W = weak, M = moderate, S = strong.

The long-term composite record displayed a seasonal cycle in monthly G/B values that was strongly influenced by the Fitzroy River discharge (Figure 2C). The highest G/B luminescence peaks corresponded to significant flood events registered at Rockhampton gauge. Moreover, the ranking of the 20 highest G/B anomaly peaks were very similar (though not identical in order) to the ranking of the 20 highest rates of stream discharge recorded during the wet season from 1921 to 2011 (Figure 3). This relationship was confirmed by the significant correlations observed between the G/B composite record (monthly and annual G/B anomalies) and all instrumental records available (water level, stream discharge, and rainfall; Table 2). The strongest relationships observed were between the annual values of G/B and the stream flow indicators (stream water level, R = 0.66, p<0.001; stream discharge, R = 0.64, p<0.001; Table 2; Figure S2). Individual G/B chronologies (GK2, SQ1, SQ2, MI1, MI2 and GK3) also showed significant correlations with the environmental data over the six different periods covered by cores (Table S4).

**Table 2:** Correlation coefficients (R) of monthly and annual G/B anomalies with environmental and climatic records.

	G/B (monthly)		G/B (annual)		PRECDS
Stream water level (m)	**0.54**	p<0.001	**0.66**	p<0.001	1922–2011*
Stream discharge(ML/day)	**0.47**	p<0.001	**0.64**	p<0.001	1922–2011**
Rainfall (mm)	**0.39**	p<0.001	**0.46**	p<0.001	1921–2011**
SOI	**0.26**	p<0.001	**0.35**	p<0.001	1921–2011**
Niño 3.4	**−0.24**	p<0.001	**−0.29**	p = 0.008	1921–2011**
PDO	**−0.38**	p<0.001	**−0.55**	p<0.001	1921–2011**
CPIPO			**−0.41**	p<0.001	1921–2004***

Bold values significant at p<0.05. Abbreviations: SOI, Southern Oscillation Index; PDO, Pacific Decadal Oscillation; CPIPO, combined paleo IPO-PDO; IPO, Interdecadal Pacific Oscillation. PRECDS, period of record for environmental and climatic data sets. * = incomplete monthly record for the period indicated. ** = complete monthly record for the period indicated. *** = only complete annual values for the period indicated. Further information on data sets is provided in Methods (section Environmental and climatic data).

**Figure 4:**
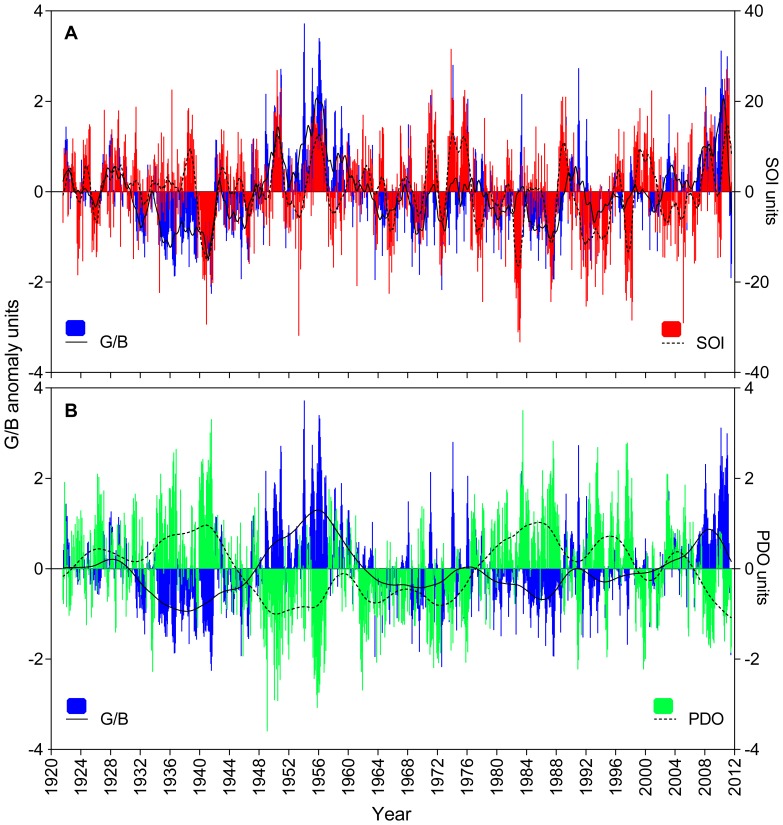
Relationship between monthly values of G/B anomalies and climatic oscillations for the period 1921–2011. (A) Southern Oscillation Index (SOI) and (B) Pacific Decadal Oscillation (PDO). Solid and dashed lines are 12-month and 85-month moving averages respectively. Data at annual resolution is provided in supplementary information (Figure S3).

**Figure 5:**
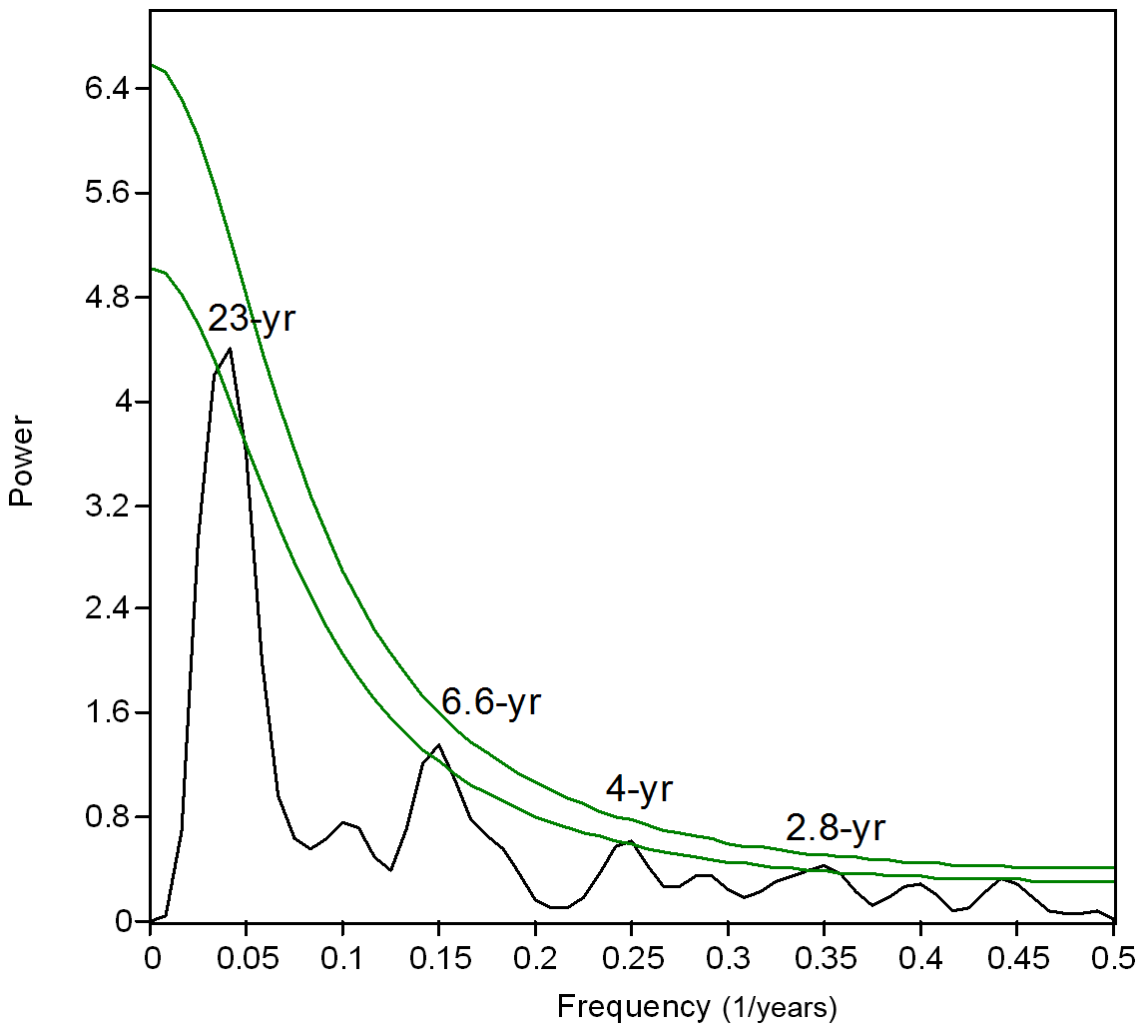
Spectral analysis (REDFIT) of annual G/B anomalies. Significant spectral peaks are indicated. Green solid lines show false-alarm levels of 95% and 99%.

The long-term composite record exhibited strong interactions with sources of inter-annual and decadal climate variability (Figures 4 and S3). While monthly G/B and SOI anomalies tended to co-vary (Figure 4A), positive and negative monthly G/B anomalies corresponded remarkably well to negative and positive PDO phases, respectively (Figure 4B). Indeed, 80% of the 20 highest G/B anomalies occurred during La Niña events (positive values of SOI) and negative PDO phases (Figure 3). Similarly, within the 20 highest records of stream discharge, 75% matched La Niña events while 60% corresponded to negative phases of the PDO (Figure 3). Correlations between monthly and annual G/B with ENSO (SOI and Niño 3.4) and PDO-IPO indices (PDO and CPIPO) showed significant relationships (Table 2). The strongest of these relationships were observed between G/B annual anomalies and the PDO index (R = −0.55, p<0.001; Table 2) and with the SOI index (R = 0.35, p<0.001; Table 2). Spectral analysis indicated cycles of 23 and 2.8–6.6 years dominated luminescence variability in the reconstructed record (Figure 5).

Correlations between G/B anomalies and environmental and climatic data (stream discharge, rainfall, SOI and PDO), using seasonal averages, revealed that the strength of relationships varied between seasons yet remained significant for most variables (Table 3). During summer (wet season), G/B anomalies showed the most robust correlations with all variables (Table 3).

**Table 3:** Correlation coefficients (R) of seasonal G/B anomalies with environmental and climatic records.

	G/B Summer		G/B Autumn		G/B Winter		G/B Spring	
Stream discharge (ML/day)	**0.69**	p<0.001	**0.58**	p<0.001	**0.44**	p<0.001	**0.29**	p = 0.007
Rainfall (mm)	**0.56**	p<0.001	**0.30**	p = 0.004	0.13	p = 0.23	0.20	p = 0.06
SOI	**0.31**	p = 0.003	0.20	p = 0.06	**0.39**	p<0.001	**0.38**	p<0.001
PDO	**−0.55**	p<0.001	**−0.43**	p<0.001	**−0.39**	p<0.001	**−0.55**	p<0.001

Bold values significant at p<0.05. Correlation for period 1921–2011. Abbreviations: SOI, Southern Oscillation Index; PDO, Pacific Decadal Oscillation. Information on data sets is provided in Methods (section Environmental and climatic data).

DISTLM analysis indicated that individually, all predictor variables (see marginal test, Table 4) explained a significant amount of the variation in the composite record, in agreement with the results obtained by correlations. Yet, the variability of the composite record was mainly explained by stream discharge and the PDO, which together accounted for 54% of the total variation (see sequential test, Table 5). While the PDO added 10% to the explained variation when stream discharge was fitted (44%), the contribution of rainfall and ENSO (SOI index) was negligible and non-significant (Table 5).

**Table 4:** Results of the marginal test performed by DISTLM-forward analysis.

Variable	SS(trace)	Pseudo-F	p	PROP
Stream discharge	26.072	67.357	0.001	**0.44**
PDO	17.771	36.834	0.001	**0.30**
SOI	7.2578	12.03	0.003	**0.12**
Rainfall	12.241	22.417	0.001	**0.20**

Significant proportions of explained variation (PROP) are given in bold.

**Table 5:** Results of the sequential test performed by DISTLM-forward analysis.

Variable	AdjustedR^2^	SS(trace)	Pseudo-F	p	PROP	CUMUL
Streamdischarge	0.4299	26.072	67.357	0.001	**0.44**	0.44
PDO	0.5283	6.1315	19.145	0.001	**0.10**	0.54
SOI	0.5235	4.78E-02	0.14762	0.689	7.99E-04	0.54
Rainfall	0.5180	8.62E-03	2.63E-02	0.882	1.44E-04	0.54

Significant proportions of explained variation (PROP) are given in bold. CUMUL = cumulative proportion of explained variation.

## Discussion

Here we provide a 90-year record of coral luminescence (G/B) based on multiple cores from the Keppel reefs, which included the 2011 flood event, one of the most significant floods recorded in the history of the southern GBR (Figure 3). Strong correlations between individual G/B records, as well as between the composite record, and each G/B series demonstrate the presence of a regional signal. Consequently, our luminescence composite record was consistent with historical flood peaks and showed significant correlations with rainfall, stream water level and discharge data. Further, DISTLM analysis revealed that the most important driver of luminescence was stream discharge as this predictor explained most of the variability in the record (44%). These linkages between luminescence and environmental variables confirmed that the Fitzroy River catchment largely influences the Keppel reefs, and therefore corals from these reefs are suitable for reconstructing regional river discharge and/or precipitation variability on monthly to decadal time scales. Similar to earlier studies from inshore reefs [16], [17], [34], we found that coral luminescence captured the variability of hydrological regimes in the southern GBR.

Spectral analysis confirmed that our composite record has cycles consistent with the typical periodicities for the PDO-IPO (15–25 years) [61], and ENSO (3–6 years) [62], yet luminescence data also indicated that river runoff in the Keppel reefs is differentially influenced by these climatic forces. Clearly, G/B variability followed the asymmetric teleconnection of ENSO with Australian climate [5], [6], [29], whereby positive G/B anomalies were commonly amplified during strong/moderate La Niña phases of ENSO, and negative G/B anomalies were occasionally enhanced during extreme El Niño events (Figure 4A). Our results, along with previous evidence on the non-stationary relationship between luminescence (runoff/rainfall) and ENSO [19], [20], confirm the widespread influence of this climatic oscillation on inshore reefs of the GBR. However, the luminescence record (G/B) consistently showed stronger correlations with the PDO than the SOI irrespective of whether monthly, seasonal or annual averages were examined. Further, DISTLM analysis indicated that the PDO explained a significant proportion (10%) of variation in luminescence, and is the only climatic variable contributing significantly to the variation explained by stream discharge. These results do not detract from the ENSO-luminescence/runoff relationship but highlight the significant role of the PDO in modulating river runoff in the Keppel reefs. A recent study documented that ENSO is not the primary source of inter-annual climate variability on the GBR and recognized that other sources such as the PDO need to be further investigated [33].

The most significant finding of our study is the relationship between coral luminescence and the PDO, which was consistently verified by both simple correlations over a range of time scales and the multivariate model. Lough [19] previously used warm and cool PDO phases as reference periods to verify links between reconstructed rainfall and river flow series based on coral luminescence and ENSO. Our study, however, is the first to directly establish the link between coral luminescence and the PDO for the southern GBR region. Further, we identified that the luminescence composite record closely mirrored the PDO index over the last century (Figures 4B and S3B). Prevalent negative G/B anomalies (reduced runoff) coincided with positive PDO phases, while periods of predominant positive G/B anomalies (increased runoff) coincided with negative PDO phases. In addition, the magnitude of G/B anomalies within these periods varied consistently with the magnitude of the PDO (Figures 4B and S3B). Therefore, our study reveals the extent of the PDO influence on the southern GBR, whilst also supporting earlier studies showing how the hydroclimatology of eastern Australia is profoundly influenced by the PDO-IPO [3]–[6], [63], [64].

Since G/B is an indicator of humic acid runoff [24], [25], its variability can also support historical analysis of human-induced erosion impacting the GBR. Decadal frequency of extreme luminescence anomalies showed that major runoff events increased after 1950 and were particularly confined to the 50′s (Figure S4), suggesting a higher influence of continental runoff (increased sediment loads) on the Keppel reefs. While instrumental data and river flow reconstructions along the GBR region show a similar variability, with increased flow conditions in the 1950s and 1970s [20], pointing to a large-scale climatic driver, the intensification of anthropogenic activities in the Fitzroy catchment by the mid-20th century [65] may also explain extreme luminescence anomalies. Historic records of human activities in the Fitzroy River catchment indicate that major shifts were related to intensive clearing of the native forests (*Acacia harpophylla*) from the 1960s to 1980s [66], increase in beef cattle numbers from 1955 [67], and expansion in coal mining since the 1970s [68]. Indeed, total suspended solids load from the Fitzroy River to the GBR is estimated to have increased 3.1×(1100–3400 ktonnes/yr) since European settlement [42]. While the length of our record (1921–2011) precludes the interpretation of luminescence changes related to European colonisation (after about 1870), the occurrence of the highest G/B peaks during the period of increased catchment modification suggests that the strong climatic signal on the luminescence record may have been enhanced by land-use changes. Increases in baseline values of runoff proxies from corals (Ba/Ca, Y/Ca) have been linked to human settlement periods and subsequent modification of river catchments by the mid to late 19th century in the central GBR [69]–[71]. Luminescence records and trace element analysis from coral cores predating European settlement are certainly required to unequivocally decouple the potential human component from the climatic signal.

### Implications of Coral Luminescence-PDO Relationship

The clear link between coral luminescence and the PDO-IPO documented here may assist studies in the historical variability of the PDO-IPO, which are critical to improving and understanding the predictability of climate change impacts in the Pacific region [58], [59], [72], [73]. Thus far, only eight paleo PDO-IPO time series of centennial scale exist [58, and references therein], and few studies have developed a reconstruction of the PDO using corals [58], [74]. Fingerprints of the PDO-IPO in corals from the South Pacific Convergence Zone [74]–[77] and the North Pacific region [78] have previously been derived from temperature/salinity proxies (δ^18^O, Sr/Ca, U/Ca). For the GBR, Calvo et al. [79] found agreement between Sr/Ca and δ^18^O records and the IPO index, Pelejero et al. [80] reported covariation of δ^11^B record (proxy for paleo-pH) and the PDO-IPO, and Lough [20] found that during the 1947–1976 PDO negative phase, the instrumental and reconstructed river flow and rainfall records were significantly correlated with ENSO indices. Thus, our study reveals a novel signature of the PDO in a non-temperature based coral proxy for the western South Pacific. While modern massive corals under the influence of rivers may contain invaluable century-length records of the PDO-IPO, fossil corals from the late Quaternary preserving luminescence lines [81] may also be useful for PDO-IPO reconstructions at millennial timescales.

The significant relationship between coral luminescence and the PDO-IPO provided insights into past disturbance regimes at the Keppel reefs. Recent studies documented extensive coral mortalities of 85% in 1991, around 40% in 2008 and 2010, and up to 100% in 2011 after severe floods from the Fitzroy River [47], [82]–[85]. Because these floods imprinted marked G/B peaks on the cores (Figure 2), they provide a basis to infer flood-induced disturbances in earlier decades prior to coral reef monitoring and instrumental records. Similarly, luminescence and runoff proxies have been used in retrospective analysis of historical disturbances and coral reef community changes on the GBR [86] Thus, based on the magnitude of G/B anomalies, we suggest that Keppel reefs likely faced coral mortality of varying extents in the wet seasons of 1951, 1954–1956, 1974 and 1983. In those years along with 1991, 2008, 2010 and 2011 (years of documented flood-induced mortality), the highest monthly discharge records of the Fitzroy River were also registered (Figure 3). Importantly, most of these events coincided with moderate-strong La Niña events during the negative phase of the PDO (Figure 3). Hence, we propose that La Niña-PDO/IPO cycles have played a significant role in the reef dynamics of the southern GBR.

## Conclusions

The luminescence record presented here displayed the temporal variability of river runoff on Keppel reefs and how such variability is influenced by the strength of La Niña and significantly modulated by the negative phase of PDO-IPO. Because recent evidence indicates that the effects of climatic phenomena (i.e. ENSO events) may vary spatially in the GBR [33], the strong relationship between PDO and coral luminescence documented here suggests that the southern GBR is particularly sensitive to the PDO influence. This implies that corals and other palaeoclimate archives from the Keppel Islands have great potential for studying the historical link between the PDO and climate variability for the southern GBR.

This study demonstrates that the PDO is a primary climatic driver of river runoff affecting the southern region of the GBR and supports growing indications that the PDO-IPO directly and indirectly influences biological communities, marine ecosystems and climate-related extreme events such as cyclones and floods/drought [31], [58], [87]–[97]. Given that emerging evidence points to a transition towards a negative PDO-IPO phase, which could increase rainfall and risk to flooding during La Niña events in Australia [32], [59], [74], we emphasize the critical role of the PDO-IPO and La Niña events in mediating disturbance and ecological processes for the GBR region in the context of rapid climate change.

## Supporting Information

Figure S1 (A) An example (core MI1) of the digital image obtained by the Spectral Luminescence Scanning (SLS) technique. The orange lines indicate transects used to extract the down-core luminescence data. (B) Monthly G/B time-series obtained from core MI1.
(TIF)

Figure S2 **Relationship between annual G/B anomalies and stream discharge rates anomalies for the period 1921–2011.** Stream discharge data from Queensland Department of Environment and Resource Management gauging stations on the Fitzroy River at The Gap (Station number 130005A) and Riverslea (130003A) (http://watermonitoring.derm.qld.gov.au/host.htm).
(TIF)

Figure S3 **Relationship between annual G/B anomalies and climatic oscillations for the period 1921–2011.** (A) Southern Oscillation Index (SOI) and (B) Pacific Decadal Oscillation (PDO). Solid and dashed lines are 6-year moving averages.
(TIF)

Figure S4 **Number of extreme G/B anomalies (>1.5 units) per decade for the period 1921–2011.**
(TIF)

Table S1 **Location and details of corals used in luminescence reconstructions from Keppel islands on the Great Barrier Reef.**
(PDF)

Table S2 **Common periods and the cores associated.**
(PDF)

Table S3 **Summary of correlation results for monthly and annual G/B anomalies among cores at different overlapping periods.**
(PDF)

Table S4 **Correlation coefficients (R) between annual G/B anomalies and environmental records, SOI and PDO for each individual coral core.**
(PDF)

Table S5 **Correlation coefficients (R) of monthly (upper) and annual (lower) G/B anomalies between cores sharing records from 1982 to 2010.**
(PDF)

Table S6 **Correlation coefficients (R) of monthly (upper) and annual (lower) G/B anomalies between cores sharing records from 1973 to 2010.** Last column includes correlation coefficients between the composite record and each core for the same period.
(PDF)

Table S7 **Correlation coefficients (R) of monthly (upper) and annual (lower) G/B anomalies between cores sharing records from 1956 to 2010.** Last column includes correlation coefficients between the composite record and each core for the same period.
(PDF)

Table S8 **Correlation coefficients (R value) of monthly (upper) and annual (lower) G/B anomalies cores sharing records from 1949 to 2010.** Last column includes correlation coefficients between the composite record and each core for the same period.
(PDF)

Table S9 **Correlation coefficients (R value) of monthly (upper) and annual (lower) G/B anomalies between cores sharing records from 1944 to 2010.** Last column includes correlation coefficients between the composite record and each core for the same period.
(PDF)
