# Supplementary figures and images for: Coral Luminescence Identifies the Pacific Decadal Oscillation as a Primary Driver of River Runoff Variability Impacting the Southern Great Barrier Reef
Source: PLoS One. 2014 Jan 8;9(1):e84305. doi: 10.1371/journal.pone.0084305 (PMC3885547; doi:10.1371/journal.pone.0084305)

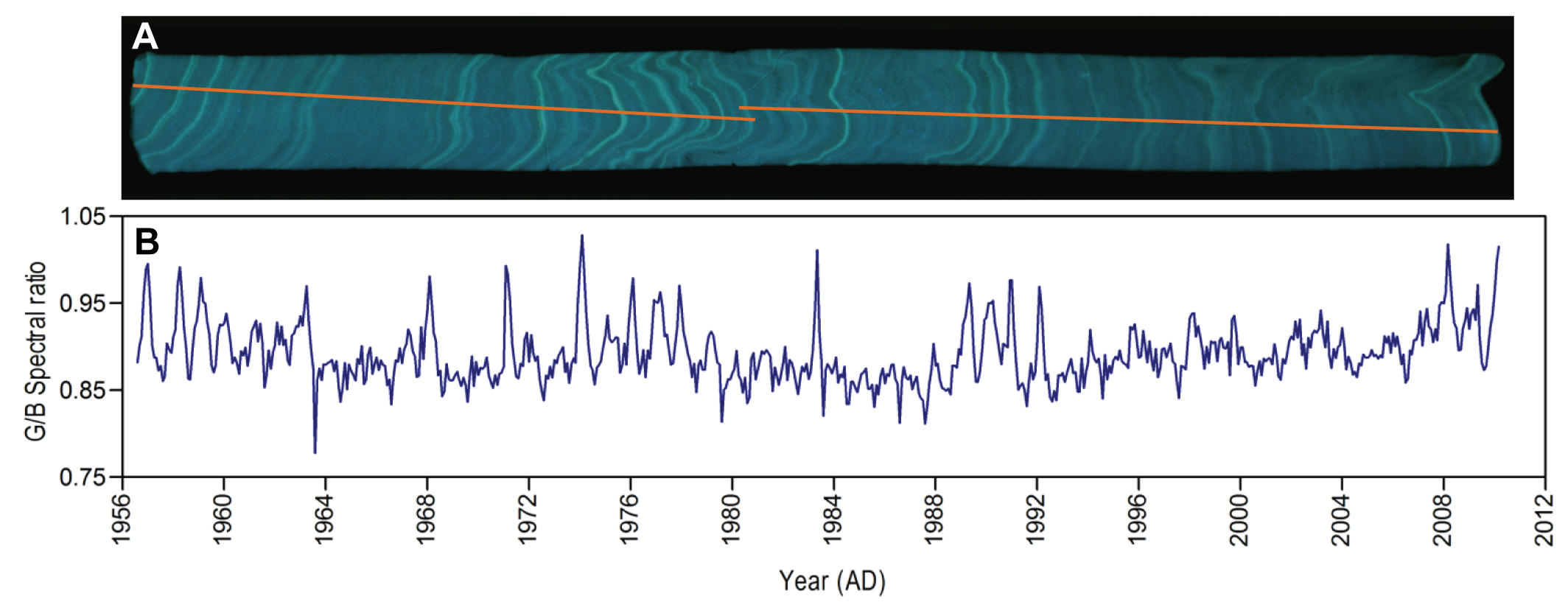

Supplement: Figure S1 — (A) An example (core MI1) of the digital image obtained by the Spectral Luminescence Scanning (SLS) technique. The orange lines indicate transects used to extract the down-core luminescence data. (B) Monthly G/B time-series obtained from core MI1. (TIF) [file pone.0084305.s001.tif]

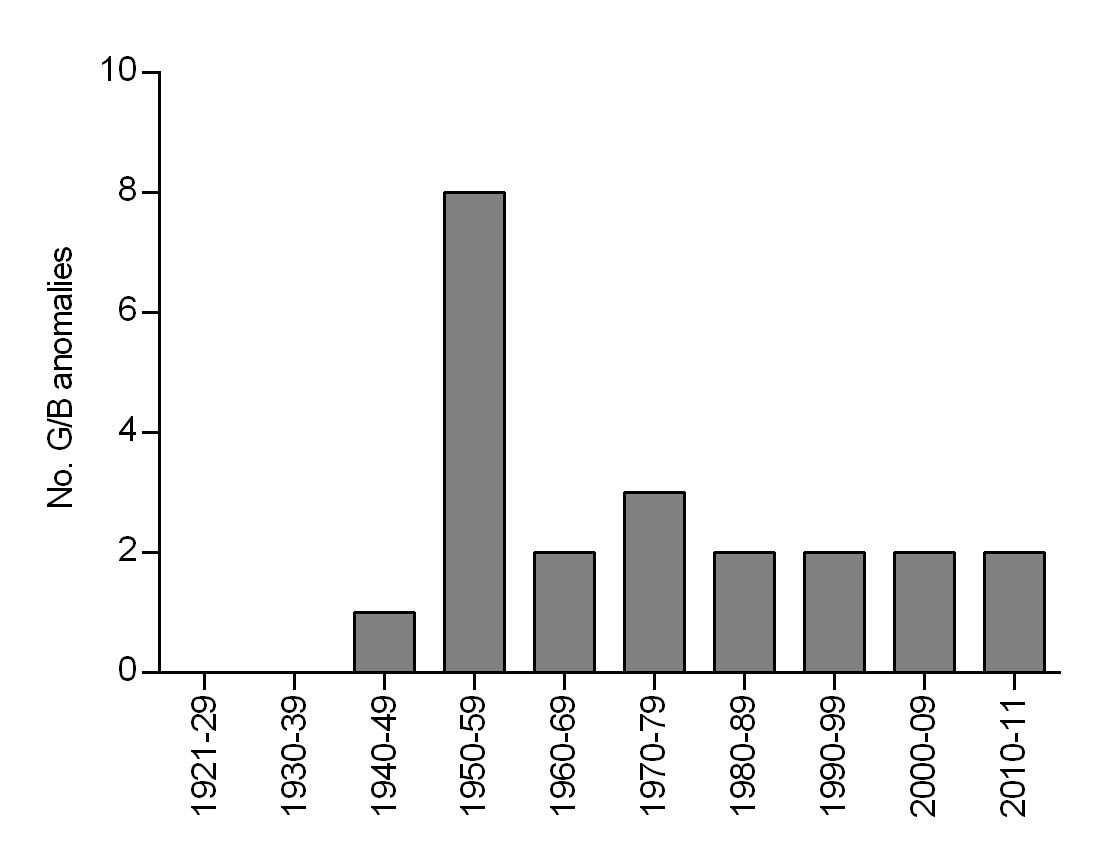

Supplement: Figure S4 — Number of extreme G/B anomalies (>1.5 units) per decade for the period 1921–2011. (TIF) [file pone.0084305.s004.tif]
