# Supplementary material for: Coral Luminescence Identifies the Pacific Decadal Oscillation as a Primary Driver of River Runoff Variability Impacting the Southern Great Barrier Reef
Source: PLoS One. 2014 Jan 8;9(1):e84305. doi: 10.1371/journal.pone.0084305 (PMC3885547; doi:10.1371/journal.pone.0084305)
Supplement: Table S1 — Location and details of corals used in luminescence reconstructions from Keppel islands on the Great Barrier Reef. (PDF) [file pone.0084305.s005.pdf]

Table S1. Location and details of corals used in luminescence reconstructions from Keppel islands on the Great Barrier Reef.

Core	Island	Location		Water depth (m)	Distance to river mouth (km)	Core length (cm)	Coral record
GK2	Great Keppel	23°09.027 S	150°58.436 E	4	42	91	1944-2010
SQ1	Square Rocks	23°05.980 S	150°53.169 E	5	47	119	1921-2010
SQ2	Square Rocks	23°05.980 S	150°53.169 E	5	47	94	1949-2010
MI1	Miall Island	23°09.300 S	150°54.209 E	3	41	64	1956-2010
MI2	Miall Island	23°09.321 S	150°54.206 E	3	41	52	1973-2010
GK3	Great Keppel	23°11.520 S	150°57.7606 E	7	38	30	1982-2012
