# Supplementary material for: Coral Luminescence Identifies the Pacific Decadal Oscillation as a Primary Driver of River Runoff Variability Impacting the Southern Great Barrier Reef
Source: PLoS One. 2014 Jan 8;9(1):e84305. doi: 10.1371/journal.pone.0084305 (PMC3885547; doi:10.1371/journal.pone.0084305)
Supplement: Table S2 — Common periods and the cores associated. (PDF) [file pone.0084305.s006.pdf]

Table S2. Common periods and the cores associated.

Common Period	Cores
1982-2010	GK2, SQ1, SQ2, MI1, MI2 and GK3
1973-2010	GK2, SQ1, SQ2, MI1 and MI2
1956-2010	GK2, SQ1, SQ2 and MI1
1949-2010	GK2, SQ1 and SQ2
1944-2010	GK2 and SQ1
