# Supplementary material for: Coral Luminescence Identifies the Pacific Decadal Oscillation as a Primary Driver of River Runoff Variability Impacting the Southern Great Barrier Reef
Source: PLoS One. 2014 Jan 8;9(1):e84305. doi: 10.1371/journal.pone.0084305 (PMC3885547; doi:10.1371/journal.pone.0084305)
Supplement: Table S3 — Summary of correlation results for monthly and annual G/B anomalies among cores at different overlapping periods. (PDF) [file pone.0084305.s007.pdf]

Table S3. Summary of correlation results for monthly and annual G/B anomalies among cores at different overlapping periods

Cores	Period	Monthly anomalies	Annual anomalies
1-6	1982-2010	All R values > 0.39 (significant at $p < 0.001$)	All R values > 0.39 (significant at $p < 0.05$)
1-5	1973-2010	All R values > 0.40 (significant at $p < 0.001$)	Most R values significant at $p< 0.05$. See exception Table S6
1-4	1956-2010	All R values > 0.37 (significant at $p < 0.001$)	All R values > 0.30 (significant at $p < 0.05$)
1-3	1949-2010	All R values > 0.50 (significant at $p < 0.001$)	All R values > 0.49 (significant at $p < 0.05$)
1, 2	1944-2010	R= 0.52 ($p < 0.001$)	R= 0.50 ($p < 0.001$)

Correlation coefficients and significance levels for each period are provided in the Tables S5 to S9. 1-6= GK2, SQ1, SQ2, MI1, MI2 and GK3; 1-5= GK2, SQ1, SQ2, MI1 and MI2; 1-4= GK2, SQ1, SQ2 and MI1; 1-3= GK2, SQ1 and SQ2; 1,2 = GK2 and SQ1.
