# Supplementary material for: Coral Luminescence Identifies the Pacific Decadal Oscillation as a Primary Driver of River Runoff Variability Impacting the Southern Great Barrier Reef
Source: PLoS One. 2014 Jan 8;9(1):e84305. doi: 10.1371/journal.pone.0084305 (PMC3885547; doi:10.1371/journal.pone.0084305)
Supplement: Table S4 — Correlation coefficients (R) between annual G/B anomalies and environmental records, SOI and PDO for each individual coral core. (PDF) [file pone.0084305.s008.pdf]

Table S4. Correlation coefficients (R) between annual G/B anomalies and environmental records, SOI and PDO for each individual coral core.

Core	Stream Water Level (m)	Stream Discharge (ML/day)	Rainfall (mm)	SOI	PDO
GK2	**0.48** ($p < 0.001$)	**0.58** ($p < 0.001$))	**0.40** ($p < 0.001$)	**0.40** ($p < 0.001$)	**-0.58** ($p < 0.001$)
SQ1	**0.63** ($p < 0.001$)	**0.54** ($p < 0.001$)	**0.40** ($p < 0.001$)	**0.24** ($p = 0.025$)	**-0.30** ($p = 0.005$)
SQ2	**0.63** ($p < 0.001$)	**0.58** ($p < 0.001$)	0.22 ($p = 0.086$)	0.17 ($p = 0.18$)	**-0.27** ($p = 0.032$)
MI1	**0.56** ($p < 0.001$)	**0.54** ($p < 0.001$)	**0.39** ($p = 0.004$)	**0.34** ($p = 0.012$)	**-0.35** ($p = 0.010$)
MI2	**0.49** ($p = 0.003$)	**0.50** ($p = 0.002$)	0.29 ($p = 0.085$)	0.27 ($p = 0.10$)	**-0.49** ($p = 0.002$)
GK3	**0.58** ($p < 0.001$)	**0.46** ($p= 0.014$)	**0.44** ($p = 0.018$)	**0.51** ($p = 0.005$)	**-0.52** ($p = 0.004$)

Significance levels in parentheses. Bold values significant at $p < 0.05$
