# Supplementary material for: Coral Luminescence Identifies the Pacific Decadal Oscillation as a Primary Driver of River Runoff Variability Impacting the Southern Great Barrier Reef
Source: PLoS One. 2014 Jan 8;9(1):e84305. doi: 10.1371/journal.pone.0084305 (PMC3885547; doi:10.1371/journal.pone.0084305)
Supplement: Table S5 — Correlation coefficients (R) of monthly (upper) and annual (lower) G/B anomalies between cores sharing records from 1982 to 2010. (PDF) [file pone.0084305.s009.pdf]

Table S5. Correlation coefficients (R) of monthly (upper) and annual (lower) G/B anomalies between cores sharing records from 1982 to 2010.

Core	SQ1		SQ2		MI1		MI2		GK3	
GK2	**0.66**	(p< 0.001)	**0.50**	(p < 0.001)	**0.71**	(p< 0.001)	**0.67**	(p < 0.001)	**0.45**	(p< 0.001)
SQ1			**0.47**	(p < 0.001)	**0.65**	(p < 0.001)	**0.70**	(p < 0.001)	**0.45**	(p < 0.001)
SQ2					**0.51**	(p < 0.001)	**0.43**	(p < 0.001)	**0.39**	(p < 0.001)
MI1							**0.74**	(p < 0.001)	**0.56**	(p < 0.001)
MI2									**0.55**	(p < 0.001)
GK2	**0.88**	(p < 0.001)	**0.48**	(p = 0.010)	**0.85**	(p < 0.001)	**0.77**	(p < 0.001)	**0.44**	(p = 0.02)
SQ1			**0.50**	(p = 0.006)	**0.88**	(p < 0.001)	**0.84**	(p < 0.001)	**0.46**	(p = 0.015)
SQ2					**0.51**	(p = 0.005)	**0.41**	(p = 0.031)	**0.39**	(p = 0.042)
MI1							**0.81**	(p < 0.001)	**0.62**	(p < 0.001)
MI2									**0.56**	(p=0.002)

Significance levels in parentheses. Bold values significant at $p < 0.05$
