# Supplementary material for: Coral Luminescence Identifies the Pacific Decadal Oscillation as a Primary Driver of River Runoff Variability Impacting the Southern Great Barrier Reef
Source: PLoS One. 2014 Jan 8;9(1):e84305. doi: 10.1371/journal.pone.0084305 (PMC3885547; doi:10.1371/journal.pone.0084305)
Supplement: Table S6 — Correlation coefficients (R) of monthly (upper) and annual (lower) G/B anomalies between cores sharing records from 1973 to 2010. Last column includes correlation coefficients between the composite record and each core for the same period. (PDF) [file pone.0084305.s010.pdf]

Table S6. Correlation coefficients (R) of monthly (upper) and annual (lower) G/B anomalies between cores sharing records from 1973 to 2010. Last column includes correlation coefficients between the composite record and each core for the same period.

Core	SQ1		SQ2		MI1		MI2		Composite record	
GK2	**0.62**	(p < 0.001)	**0.47**	(p < 0.001)	**0.72**	(p < 0.001)	**0.66**	(p < 0.001)	**0.76**	(p < 0.001)
SQ1			**0.45**	(p < 0.001)	**0.61**	(p < 0.001)	**0.64**	(p < 0.001)	**0.68**	(p < 0.001)
SQ2					**0.48**	(p < 0.001)	**0.4**	(p < 0.001)	**0.59**	(p < 0.001)
MI1							**0.73**	(p < 0.001)	**0.80**	(p < 0.001)
MI2									**0.75**	(p < 0.001)
GK2	**0.79**	(p < 0.001)	**0.33**	(p = 0.049)	**0.85**	(p < 0.001)	**0.77**	(p < 0.001)	**0.80**	(p < 0.001)
SQ1			**0.46**	(p = 0.005)	**0.79**	(p < 0.001)	**0.80**	(p < 0.001)	**0.82**	(p < 0.001)
SQ2					**0.35**	(p = 0.033)	0.26	(p = 0.12)	**0.44**	(p= 0.007)
MI1							**0.80**	(p < 0.001)	**0.86**	(p < 0.001)
MI2									**0.80**	(p < 0.001)

Significance levels in parentheses. Bold values significant at p < 0.05
