# Supplementary material for: Coral Luminescence Identifies the Pacific Decadal Oscillation as a Primary Driver of River Runoff Variability Impacting the Southern Great Barrier Reef
Source: PLoS One. 2014 Jan 8;9(1):e84305. doi: 10.1371/journal.pone.0084305 (PMC3885547; doi:10.1371/journal.pone.0084305)
Supplement: Table S8 — Correlation coefficients (R value) of monthly (upper) and annual (lower) G/B anomalies cores sharing records from 1949 to 2010. Last column includes correlation coefficients between the composite record and each core for the same period. (PDF) [file pone.0084305.s012.pdf]

Table S8. Correlation coefficients (R value) of monthly (upper) and annual (lower) G/B anomalies cores sharing records from 1949 to 2010. Last column includes correlation coefficients between the composite record and each core for the same period.

Core	SQ1		SQ2		Composite record	
GK2	**0.54**	($p < 0.001$)	**0.63**	($p < 0.001$)	**0.74**	($p < 0.001$)
SQ1			**0.50**	($p < 0.001$)	**0.80**	($p < 0.001$)
SQ2					**0.84**	($p < 0.001$)
GK2	**0.53**	($p = 0.004$)	**0.67**	($p < 0.001$)	**0.77**	($p < 0.001$)
SQ1			**0.49**	($p < 0.001$)	**0.64**	($p < 0.001$)
SQ2					**0.76**	($p < 0.001$)

Significance levels in parentheses. Bold values significant at $p < 0.05$
