# Supplementary material for: Coral Luminescence Identifies the Pacific Decadal Oscillation as a Primary Driver of River Runoff Variability Impacting the Southern Great Barrier Reef
Source: PLoS One. 2014 Jan 8;9(1):e84305. doi: 10.1371/journal.pone.0084305 (PMC3885547; doi:10.1371/journal.pone.0084305)
Supplement: Table S9 — Correlation coefficients (R value) of monthly (upper) and annual (lower) G/B anomalies between cores sharing records from 1944 to 2010. Last column includes correlation coefficients between the composite record and each core for the same period. (PDF) [file pone.0084305.s013.pdf]

Table S9. Correlation coefficients (R value) of monthly (upper) and annual (lower) G/B anomalies between cores sharing records from 1944 to 2010. Last column includes correlation coefficients between the composite record and each core for the same period.

	SQ1		Composite record	
GK2	**0.52**	(p < 0.001)	**0.69**	(p < 0.001)
SQ1			**0.60**	(p < 0.001)
GK2	**0.50**	(p < 0.001)	**0.69**	(p < 0.001)
SQ1			**0.60**	(p < 0.001)

Significance levels in parentheses. Bold values significant at p < 0.001
